# Effect of Cavity Design on the Strength of Direct Posterior Composite Restorations: An Empirical and FEM Analysis

**DOI:** 10.1155/2011/214751

**Published:** 2011-12-15

**Authors:** V. Susila Anand, C. Kavitha, C. V. Subbarao

**Affiliations:** ^1^Conservative Dentistry and Endodontics, Saveetha Dental College, Saveetha University, Madras 600077, India; ^2^Bioengineering Laboratory, Engineering Design Department, Indian Institute of Technology Madras, Madras 600036, India

## Abstract

The aim of the present study was to verify the hypothesis that cavity design does not affect the strength of direct composite restorations as do material properties. Finite element modeling (FEM) and empirical testing were done for two cavity designs: a box shape (cube) and a concave shape (U). Two microhybrid composites were used to prepare the samples with the help of split stainless steel moulds. Compressive strength was tested. The results were statistically analyzed. Both FEA and empirical testing were complementary to each other in that the concave shape showed a significantly higher strength than box. Material properties affected the values only when box shape was used. The null hypothesis is thus rejected, and it is concluded that design significantly affects the strength of direct composite restorations.

## 1. Introduction

Composite restorations have revolutionized restorative dentistry due to their conservative nature, adhesive bonding and patient appeal. The adhesive bonding, ability makes it unnecessary to remove tooth structure for retention, prevention, and convenience. Successful restorations can be done with less precise preparations.

With paradigm shift from retentive restorations to conservative restorations, there is an increasing emphasis on minimally invasive cavity preparations. There are many studies reporting the effect of cavity design on the fracture resistance of teeth restored with indirect composite restorations [1]. Other studies [2, 3] have evaluated the effect of cavity design on the marginal leakage of composite restorations. One of the earliest applications of engineering principles in cavity design, with the objective of minimizing stress concentration was by Bronner [4]. Further work was subsequently reported by Gabel [5], Brown [6], and Weiland [7]. The conclusions of these studies were that the cavity preparation should enhance the properties of the restorative material in such a way that those unfavorable are properly compensated for in terms of stresses; the cavity preparation has to allow the operator to work efficiently in such a way that a mechanically sound preparation is obtained [8].

Noonan [9] provided one of the first studies of cavity design utilizing two-dimensional photoelastic stress analysis. From this followed a great number of studies using the photoelastic technique [10–13] that investigated the effect of differing Class II cavity designs on the stress dissipation with the remaining tooth structure and the restorative material.Granath and Hiltscher[14] complemented his analog studies by strain gauge measurements when investigating the effect of the buccolingual shape, while Fisher and Caputo [15] investigated both extracoronal and intracoronal preparations. The finite element method was first introduced into the area of stress analysis of biological structures in 1972 by Brekelmans et al. [16]. But there is a dearth in studies on the effects of cavity design on the strength of direct composite restorations.

The aim of the present study is to evaluate the effect of cavity design on the strength of direct composite restorations. For this, we propose a null hypothesis that cavity design does not affect the strength of direct composite restorations as do the material properties. To validate this hypothesis, two cavity preparation designs, a conventional box and a new minimally invasive concave shaped cavity with 4° taper, were studied with two composite restorative materials, Restofill and Esthet-X.

## 2. Materials and Methods

**Figure 1:**
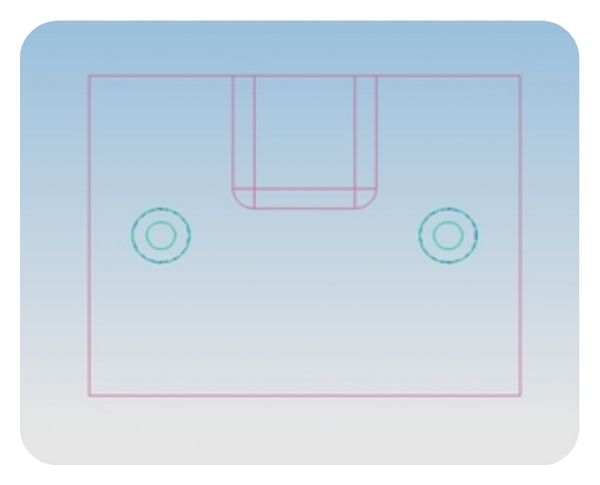
2D image of s.s mould prototype—box design.

**Figure 2:**
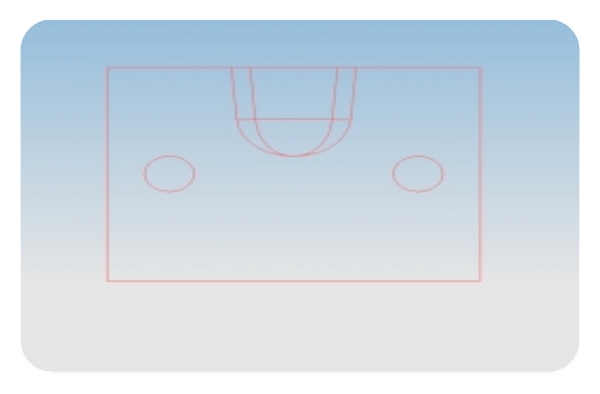
2D image of s.s mould prototype—concave design.

Two split stainless steel (s.s) moulds with suitable plungers were fabricated using prototype designing with Pro E software. The moulds were made of two different shapes, box (cube—group I; Figure 1) and concave shape (U design with 4° taper—group II; Figure 2). The internal line and point angles were rounded and the dimensions were 10 mm × 10 mm × 10 mm (height × width × length). The internal radius of “U” in concave shape was 5 mm. The inner surfaces of the moulds were smooth and well polished and millimeter markings were inscribed on one wall to facilitate incremental composite curing. 

Two microhybrid composites, Restofill (subgroup A) and Esthet-X (subgroup B) were used in the study. The composition of the materials is given in Table 1. Twenty samples were prepared for each design, 10 for each material. The material was condensed into the moulds using Teflon-coated composite condensers and pressure was applied to remove voids with the help of the built in s.s plunger of the mould. Every 2 mm increment of composite was cured with QTH curing light with an intensity of 400 mW/cm^2^ for 40 s. The four corners of the moulds were focused for 10 s each with the curing tip to ensure adequate curing. The final increment was covered with a mylar strip during curing.

The split mould design ensured that the specimens were retrieved easily without stress. 

### 2.1. Empirical Testing

The composite samples were loaded under compression in a universal testing machine (Instron) at a cross-head speed of 1 mm/min. The load at fracture was noted and the compressive strength calculated.

### 2.2. FEM

**Figure 3:**
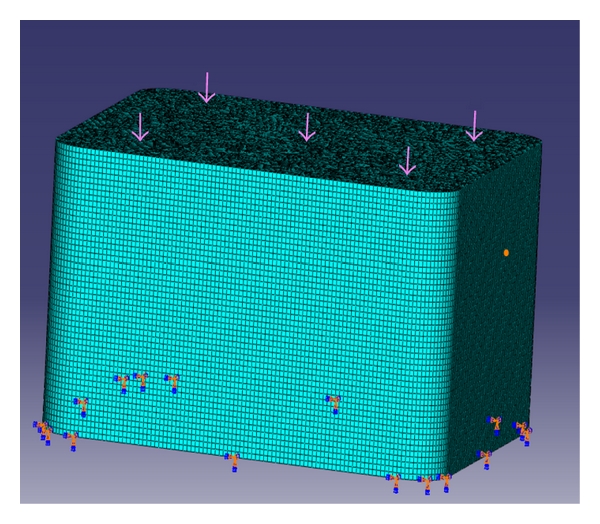
Cube mesh model.

**Figure 4:**
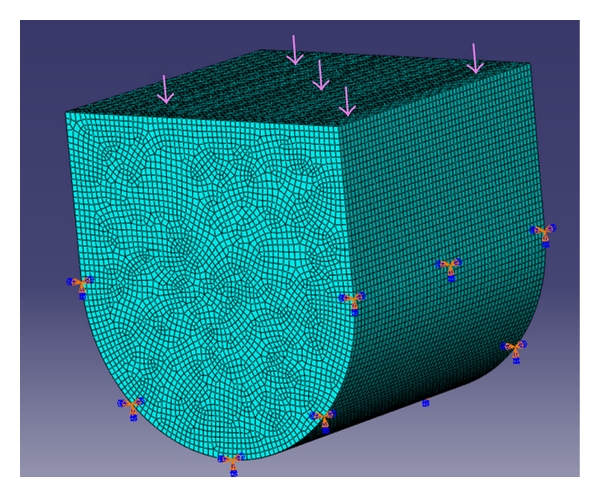
U-shaped mesh model.

Abaqus 6.7 (finite element tool) was used to create the 3-dimensional models of the above moulds using high-resolution images. A cube and a U-shaped model with10 × 10 × 10 mm dimensions were designed. (Figures 3 and 4) The internal radius of “U” was 5 mm. The models had the following physical characteristics: elastic modulus of 20 GPa, Poisson's ratio (*γ*) of 0.3, and density of 700 kg/m^3^. Dynamic explicit procedure type was chosen for finite element analysis with encastre boundary conditions. A mesh size of 1 mm and an element size of 0.1 mm were used for the study. The von Mises stresses were calculated at 1500 N load with the Abaqus 6.7 EF1 software.

### 2.3. Statistical Analysis

*t*-test was done to analyze the statistical significance of the results. Levene's test of Equality of variances and *t*-test for equality of means was done for intra- and intergroup comparisons of strength values.

## 3. Results

### 3.1. Empirical Testing

The compressive strength was found to be 33 MPa (group I A box design, Restofill), 21.25 MPa (group I B Box design, Esthet-X), 49 MPa (group II A conservative design Restofill), and 49.49 MPa (group II B concave design Esthet-X; Table 2).

### 3.2. FEM

**Figure 5:**
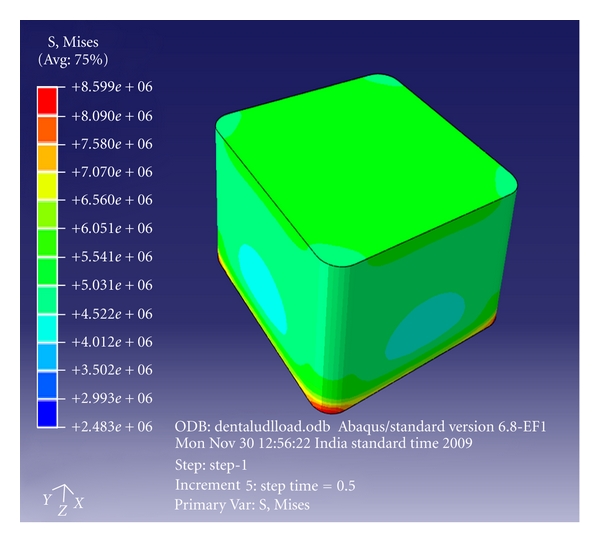
Finite element analysis (FEA) of cube model—box-shaped cavity design.

**Figure 6:**
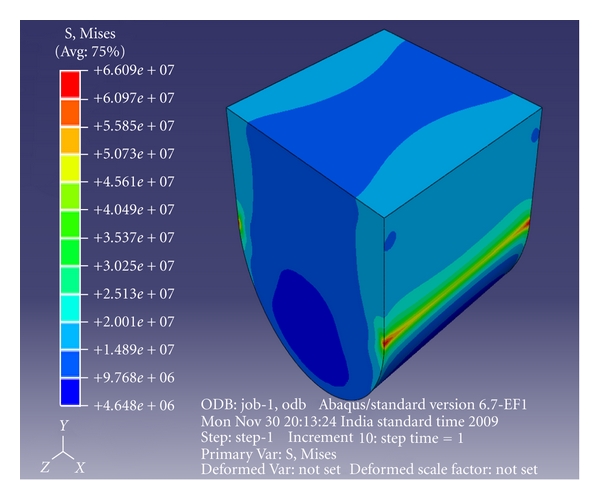
FEA of U-shaped model—concave cavity design.

The von Mises stress for group I (cube mesh) was found to be 25.6 MPa (Figure 5) and for group II (U-shaped model) 66 MPa (Figure 6) at a load of 1500 N. There was 59% improvement in the strength when the concave design was used (Table 3).

Empirical test showed statistically significantly greater strength for group II than group I (*P* = 0.001; Table 2) The results of FEM also showed greater values for Group II than I (Table 3). Among subgroups, A had significantly greater strength (Table 2) than B when box cavity (group I) was used (*P* = 0.001). When concave-shaped cavity was used, there was no difference in the strength values between the 2 materials (*P* ≥ 0.05). When the concave design was used subgroup A showed 33% and subgroup B showed 57% improvement in the strength. 

## 4. Discussion

The present study proposed a null hypothesis that cavity design does not play a significant role on the strength of direct composite restorations. Based on this, 2 cavity designs were tested. One of the first studies on the effect of cavity preparation on the strength of teeth was by Vale [17]. Effect of width of isthmus preparation was studied and it was found that there was no difference in the ultimate fracture strength of the sound teeth with the 1/4 intercuspal distance cavity preparation. However, when the isthmus width was increased from 1/4 to 1/3 intercuspal distance, there was a significant weakening of the prepared tooth [8].

A study on two-stage shape optimization process for cavity preparation found that the stress level at the tooth-restoration interface in the optimized design was reduced significantly compared with the conventional design, irrespective of the bonding condition [18]. In the present study two cavity designs were evaluated for their effect on the strength of two different composite restorative materials. It was found that the conservative design (concave shape) had better strength (twofold on an average) than conventional design irrespective of the type of material. The greatest improvement in strength was noticed for subgroupB (Esthet-X) during empirical testing when the cavity design was altered (concave versus box). The conventional design (box shape) showed that Restofill had significantly better strength than Esthet-X (1- and 1/2-fold).

Cavities with large cavosurface angle (CSA), which are considered to lead to low stress concentrations near the free surface, were found to have better marginal integrity in a finite element study using axisymmetric models [19]. Similar cavity shapes were also investigated by Hembree Jr. [20]. A saucer-shaped cavity design was proposed and investigated for composite restorations [21–23]. In a clinical trial, 51 such preparations were completed and evaluated annually for 10 years and 70% of the restorations were acceptable for continued use [22]. A more recent study compared the survival of restorations placed in saucer-shaped cavities to that of restorations placed in tunnel preparations [23]. After a mean service life of 28.8 and 30.3 months, the proportion of the tunnel and saucer-shaped restorations survived was 46% and 76%, respectively. Moreover, saucer-shaped restorations showed lower caries development than the tunnel restorations after an observation period of 24 months. The alternative cavity shapes proposed in the above works were mostly based on experience and intuition of the investigators [18]. In the present study finite element modelling (FEM) and empirical testing showed that the conservative concave design, which had a larger CSA due to the 4° taper, demonstrated a two-and-half-fold increase in strength than the conventional box design which had a butt joint and hence lower CSA. Thus the results of the present agree with those of the above workers.

 In another study using shape optimization technique, it was found that the optimized design showed significant (24%) improvement in the resistance against debonding under compressive load compared with the traditional design [24]. In the present study, the concave design (group II) showed 59% and 57% increase in compressive strength compared to conventional box design (group I) in FEM and empirical testing, respectively.

In a study on mechanics of fracture of enamel and dentin it was found that they behaved as brittle materials and that forces generated through mastication and bruxism had the potential to initiate fractures when the tooth had been altered by cavity preparation [25]. Therefore, the importance of designing the cavity preparation to reduce any potential stress concentrations was recognized. That radical removal of tooth structure results in reduction of fracture strength of teeth, and increased marginal gap formation has been well documented in many other studies [1, 26–29].

The results of the present study are in agreement with the above studies and the conservative design resulted in a stronger restoration. Even a material with a lower strength could perform equally well as a material with higher strength, if a conservative design is chosen for placing the restoration.

Hence the null hypothesis that preparation design does not affect the strength of direct composite restorations is rejected.

## 5. Conclusions

The present study tested the effect of cavity designs on only the compressive strength of direct composite restorations. The tensile and flexural strengths were not analyzed. Also only 2 composite materials were tested. Nevertheless the importance of conservative design has been highlighted by the findings of this study. Hence within the limitations of this invitro study, it may be concluded that a conservative concave cavity design with a 4^°  ^taper provides a significantly higher strength to direct composite restorations irrespective of the restorative material used. Such a cavity design also fulfills the objectives of minimally invasive dentistry. Hence the null hypothesis that design does not affect the strength of the direct composite restorations is rejected. However, further FEM analyses and clinical investigations of the other conservative cavity designs are needed to validate the alternate hypothesis that cavity design plays a significant role in improving the tensile and flexural strength of direct composite restorations.

## Figures and Tables

**Table 1:** Composition of composite materials used.

Esthet-X (DENTSPLY)	Restofill (Anabond Stedman)
Dimethacrylate resins	Bis-GMA
Urethane modified Bis-GMA	TEGDMA
Barium boroaluminosilicate glass	Barium boroaluminosilicate glass
Barium fluoroaluminosilicate glass	

**Table 2:** Results of FEM.

Group	Shape	Strength (MPa)	% improvement
I	Box	25.6	59
II	Concave	66	

**Table 3:** Strength of experimental groups.

Group	Sub-group	*n*	Mean Strength (MPa)	Standard deviation	% improvement
I	A	10	33	2.25918	33
	A	10	49	2.34434	
II	B	10	21.25	2.79466	57
	B	10	49.49	3.48038	
